# Evidence of the Long-Term Protective Effect of Moderate-Intensity Physical Activity on Cognitive Function in Middle-Aged and Elderly Individuals: A Predictive Analysis of Longitudinal Studies

**DOI:** 10.3390/life14101343

**Published:** 2024-10-21

**Authors:** Zikang Hao, Xianliang Zhang, Yu Wang

**Affiliations:** 1 School of Physical Education, Shandong University, Jinan 250061, China; 2 Exercise Science Laboratory, Department of Physical Education, Ocean University of China, Qingdao 266005, China; 3 Department of Physical Education, Moscow State University of Sport and Tourism, Kirovogradskaya Street, 21, Moscow 117519, Russia

**Keywords:** physical activity, cognitive decline, dementia prevention, machine learning, trend projection

## Abstract

Objective: To investigate the effects of different intensities of physical activity (PA) on cognitive function in middle-aged and elderly individuals, and to predict future trends in cognitive ability using longitudinal data to assess the long-term role of PA in cognitive preservation. Methods: Data from the China Health and Retirement Longitudinal Study (CHARLS) were utilized. Mixed-effects models were employed to analyze the impacts of low-intensity PA (LPA), moderate-intensity PA (MPA), and vigorous-intensity PA (VPA) on overall cognition, episodic memory, and mental intactness. Random forest and XGBoost machine learning methods were employed to further validate the effects of PA. ARIMA models predicted future cognitive trends under the influence of PA. Results: MPA demonstrated significant advantages in preserving cognitive function, particularly in overall cognition and episodic memory. While LPA had some protective effects, they were less significant than those of MPA, and VPA did not show advantages. Machine learning methods confirmed these findings. ARIMA model predictions indicated that the protective effects of MPA on cognitive function are likely to persist in the future. Conclusions: Moderate-intensity physical activity is associated with the preservation of cognitive ability in middle-aged and elderly individuals and may continue to provide this benefit in the future; however, further in-depth research is needed for confirmation.

## 1. Introduction

As the global population ages, cognitive decline among the elderly has become a serious public health issue [1]. It is not only an early sign of dementia but also increases the risk of mortality and disability, imposing a substantial economic burden on society [2]. Statistics indicate that by 2015, the global economic cost of dementia was approximately USD 957.56 billion, projected to rise to USD 2.54 trillion by 2030 and reach USD 9.12 trillion by 2050 [3]. In China, with its significant aging population, individuals aged 65 and over numbered 207 million by 2021, accounting for 14.2% of the total population. Among these, about 15–20% are diagnosed with mild cognitive impairment (MCI), and 5–7% have dementia [4]. Therefore, effectively preventing cognitive decline to delay the onset of dementia holds great public health significance.

Proactive interventions before the progression to dementia—such as physical activity (PA), cognitive training, and dietary improvements—may delay further deterioration of cognitive function, thereby reducing dementia risk [5,6]. Numerous studies have explored the relationship between PA and cognitive function, particularly in the elderly. Multiple meta-analyses and systematic reviews have indicated that PA is significantly associated with a lower risk of cognitive decline and dementia. For example, a longitudinal meta-analysis by Beckett et al. demonstrated that PA can significantly reduce the incidence of Alzheimer’s disease [7]. Guure et al. further found that PA has protective effects against all-cause dementia and Alzheimer’s disease [8], while Iso-Markku et al. confirmed these results remain robust over long-term follow-up [6]. However, not all studies have consistently validated the benefits of PA. Sabia et al.’s prospective longitudinal study in the Whitehall II cohort did not find a significant association between PA and cognitive decline or dementia [9].

These discrepancies may be related to the dose–response effect of PA as an intervention. The intensity, duration, and frequency of PA collectively determine its impact on cognitive function [10,11]. Many studies have not distinguished between different PA intensities, treating it as a single variable and overlooking potential differences among low-, moderate-, and high-intensity PA [12,13]. Moreover, most existing research employs cross-sectional designs, revealing only immediate associations between PA and cognitive function without adequately demonstrating long-term dynamic changes [14,15]. Critically, few studies have attempted to predict the long-term impact of PA on future cognitive trends, which is essential for formulating effective and sustained prevention strategies. It is also important to note that the type of physical activity (e.g., aerobic, resistance, or mixed) [16] or the content of the activity (such as dual-task exercises incorporating cognitive training or meditation-based activities involving mental load) [17] can likewise influence outcomes differently.

Based on this, this study adopts a longitudinal design to conduct a targeted and detailed analysis of the impact of different PA intensities on cognitive function in middle-aged and older adults. The evaluation of cognitive abilities was performed based on three tests: episodic memory, mental intactness, and total cognitive abilities. Physical activity intensity was differentiated based on the IPA Q-SF questionnaire. Machine learning methods were employed to validate the robustness of these effects, while predictive models were used to explore the long-term influence of PA on future cognitive changes. In the area of PA intensity, the findings aim to provide scientific evidence and guidance for developing strategies to prevent cognitive decline.

## 2. Materials and Methods

### 2.1. Dataset and Study Design

This study utilized data from the CHARLS, a publicly available database. CHARLS is a large-scale, nationwide prospective cohort study that conducted sampled surveys in over 30 cities across China. Starting in 2011, surveys were administered every 2–3 years until 2020, encompassing five waves (2011–2012, 2013–2014, 2015–2015, 2018, 2020). The original study design and details are accessible online at http://charls.pku.edu.cn/. The CHARLS was approved by the Institutional Review Board of Peking University (IRB00001052-11015), and written informed consent was obtained from all participants prior to inclusion [18].

**Figure 1:**
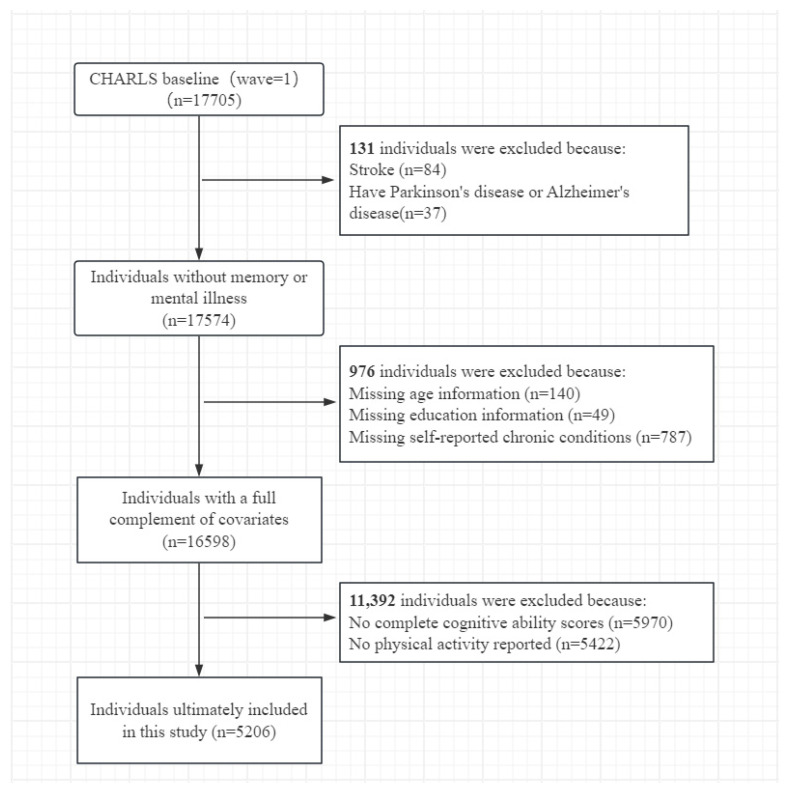
Flowchart of the participant selection process in this study.

Based on data completeness and other factors, we screened the baseline sample of 17,705 participants and ultimately included data from 5206 participants for subsequent analysis. The exclusion process is illustrated in Figure 1.

### 2.2. Cognitive Ability Assessment

Following standard database protocols and prior studies, cognitive performance was assessed using two key measures: episodic memory and mental intactness. Episodic memory evaluates an individual’s capacity to recall ten recently presented Chinese words, both immediately (immediate recall) and after a four-minute delay (delayed recall). The episodic memory score represents the average of these two recall tasks, with a range of 0 to 10 [19].

Mental intactness evaluates cognitive function using specific items from the Telephone Interview for Cognitive Status (TICS), such as serial subtraction of 7 from 100 up to five times, orientation to date—including month, day, year, and season—identifying the day of the week, and the ability to replicate a displayed figure. Scores from these tasks generate a mental intactness score between 0 and 11. The total cognitive score, ranging from 0 to 21, is the sum of the episodic memory and mental intactness scores [20].

Specifically, episodic memory is the ability to recall memories of specific events or experiences, especially time, place, and situation. This form of memory is closely linked to the hippocampus and the frontal lobes of the brain. Episodic memory is particularly important for the middle-aged and elderly population, as it is one of the earliest cognitive functions to become problematic in the elderly, and is especially an early marker of cognitive degenerative diseases such as Alzheimer’s disease. Mental integrity is a comprehensive indicator for assessing overall cognitive health, covering multiple dimensions such as executive function, attention, and language comprehension. It reflects an individual’s overall competence in cognitive health and is key to assessing cognitive abilities in everyday life. Cognitive decline typically manifests as a gradual decline in multiple cognitive domains, not just changes in memory capacity or a single cognitive domain. By assessing overall cognitive performance, the full range of cognitive changes can be better captured, leading to a more comprehensive understanding of the impact of physical activity on cognitive health.

### 2.3. Physical Activity Assessment

PA and its intensity were quantified using the International Physical Activity Questionnaire Short Form (IPAQ-SF). Participants reported the number of days and typical daily duration they engaged in three types of activities—light, moderate, and vigorous—during a typical week. Engaging in “light”, “moderate”, or “vigorous” PA was defined as participating in the corresponding activity type for at least 10 min per session on 3 or more days per week. Participants were categorized into PA intensity levels of “none”, “light”, “moderate”, or “vigorous” based on the highest intensity they reported [21].

### 2.4. Covariates

Demographic factors such as age, gender, residence, educational level, and marital status, along with self-reported chronic disease status (including hypertension, diabetes, cancer, lung disease, heart disease, arthritis, dyslipidemia, liver disease, kidney disease, gastrointestinal disease, and asthma), were included in the analysis. Age was categorized into “over 60” and “45–60” years. Residence was classified as “rural” or “urban”. Educational levels were divided into “below primary school”, “primary school”, “middle school”, and “high school or above”. Marital status was categorized as “married” or “other”. Chronic disease status was classified as “present” or “absent”.

### 2.5. Statistical Analysis

To explore the relationship between PA intensity and cognitive ability, as well as the interaction between PA intensity and time, we employed mixed-effects models with time interaction terms. The ability of the model to handle both time-invariant variables (e.g., gender, baseline age) and time-varying variables (e.g., motor level, cognitive ability) facilitates the study of complex time-dynamic interactions [22]. Specifically, we constructed models and incrementally introduced covariates:

- Model 1: Included only the independent variable (PA intensity) and the dependent variable (cognitive ability) to understand the basic impact of PA intensity.
- Model 2: Added demographic variables such as gender, age, and educational level to Model 1 to control for their influence and further assess their effects on cognitive ability.
- Model 3: Incorporated health-related factors (e.g., chronic disease status, smoking, alcohol consumption) into Model 2 for a more comprehensive analysis of health status on cognitive ability.

Each model was compared using the Akaike Information Criterion (AIC) and log-likelihood values to determine the best fit. We then introduced time interaction terms to examine whether the effect of PA intensity on cognitive ability changes over time. The models reported β coefficients, 95% confidence intervals (CIs), and *p*-values to assess the relationship between PA intensity and cognitive ability.

Secondly, to validate the effectiveness and robustness of the results, we employed two machine learning methods: random forest and extreme gradient boosting (XGBoost) [23].

- Random forest: Used to explore the influence and importance of various variables, especially PA intensity, on cognitive ability. The model included PA intensity and covariates such as age, educational level, and health status to predict cognitive outcomes. The original dataset was split into training and test sets in a 7:3 ratio. Variable importance analysis determined which factors contributed most to predicting changes in cognitive ability. Model performance was evaluated using mean squared error (MSE); lower MSE values indicate better performance.
- XGBoost: Applied to further optimize predictive modeling of cognitive ability. Hyperparameters were tuned through grid search and cross-validation to enhance model performance. Both training and test sets’ root mean squared error (RMSE) and MSE were used to assess performance; lower values indicate higher predictive accuracy and better model fit.

Finally, to investigate our hypothesis regarding future trends—that PA intensity will continue to protect cognitive ability—we applied an Autoregressive Integrated Moving Average (ARIMA) model. The model analyzes historical trends and patterns in time series, which captures changes in cognitive ability over time and provides long-term forecasts of future changes, and can be flexibly adapted to a variety of time series patterns based on data characteristics. Each participant’s cognitive scores across different waves were modeled to predict future performance. The model was adjusted based on the periodicity and trend characteristics of the data. Model fit and predictive accuracy were evaluated using the Ljung–Box *p*-value; a value greater than 0.05 indicates good performance with high predictive accuracy [24]. All statistical analyses were performed using the R software package (version 4.4.1, R Foundation for Statistical Computing, Vienna, Austria). The following R packages were utilized to implement machine learning models and data visualization: “ggplot2”, “xgboost”, “caret”, “dplyr”, and “haven”.

### 2.6. Data Availability

Qualified researchers can freely access the CHARLS database online at https://charls.pku.edu.cn/ under a data use agreement. The dataset used in this study is available from the first author via email upon reasonable request.

## 3. Results

### 3.1. Baseline Characteristics of Participants

Among the 5206 individuals included in this study, 1777 were classified as engaging in vigorous-intensity physical activity (VPA), 1621 in moderate-intensity physical activity (MPA), and 1300 in low-intensity physical activity (LPA), and 508 reported no physical activity. The sample consisted of 51.5% females and 48.5% males; 89.2% were married, while 10.8% had other marital statuses. Detailed characteristics are presented in Table 1.

### 3.2. Results of Multistep Regression Analysis of PA Intensity on Cognitive Ability

In the unadjusted model (Model 1), both LPA and MPA were significantly associated with higher total cognitive scores (LPA: β = 0.271, 95% CI: 0.178–0.912, *p* = 0.004; MPA: β = 0.486, 95% CI: 0.514–1.224, *p* < 0.001), while the effect of VPA was not statistically significant (*p* = 0.267). Similar patterns were observed for episodic memory and mental intactness, with MPA showing a stronger positive effect.

After adjusting for demographic factors such as gender, age, and education (Model 2), LPA and MPA continued to exhibit significant positive associations with episodic memory and mental intactness (LPA on episodic memory: β = 0.216, 95% CI: 0.005–0.457, *p* = 0.048; MPA on mental intactness: β = 0.707, 95% CI: 0.117–0.741, *p* = 0.007). The effect of VPA remained non-significant after adjustment.

**Figure 2:**
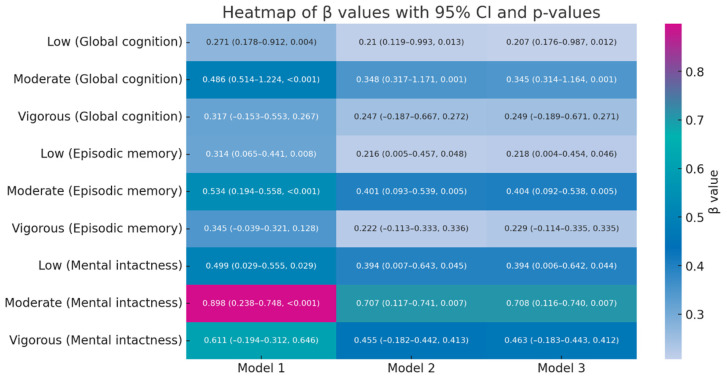
Heatmap of multistep regression analysis results of PA intensity on cognitive ability. Each cell represents the β coefficient, 95% confidence interval, and *p*-value for the association between PA intensity and cognitive ability in a specific model. The color gradient indicates the magnitude and direction of the β value; colors closer to red signify larger positive β values, while colors closer to blue indicate smaller or negative β values.

Further adjustment for health-related factors (Model 3) did not alter the significant positive impact of LPA and MPA on cognitive ability. Notably, MPA continued to show a strong positive effect on episodic memory and mental intactness (MPA on mental intactness: β = 0.708, 95% CI: 0.116–0.740, *p* = 0.007). The effect of VPA remained non-significant. Detailed results are presented in Figure 2 and Supplementary Table S1.

Comparisons of Akaike Information Criterion (AIC) and log-likelihood values across models indicated that the overall fit improved as covariates were progressively added. Importantly, the β coefficients for MPA remained significant across all models, demonstrating a substantial cognitive protective effect.

### 3.3. Impact of the Interaction between PA Intensity and Time on Cognitive Ability

**Figure 3:**
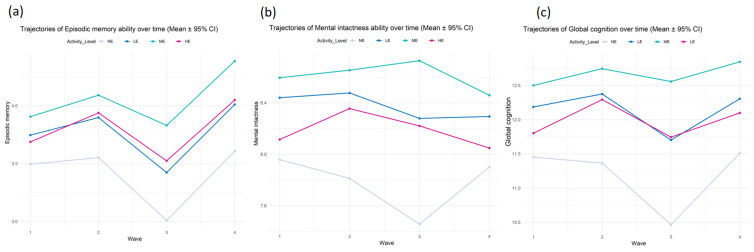
Impact of the interaction between PA intensity and time on cognitive ability. (**a**) Effect on episodic memory; (**b**) effect on mental intactness; (**c**) effect on total cognitive ability.

As shown in Figure 3a and Supplementary Table S2, MPA exhibited a sustained significant positive effect on episodic memory over time, particularly at Wave 3 (β = 0.682, *p* = 0.001). LPA also showed a significant improvement at Wave 3, but the effect of VPA diminished at Wave 4 (*p* = 0.166).

In the analysis of mental intactness (Figure 3b), both LPA and MPA displayed consistent positive effects over time. MPA had the most significant enhancement at Wave 3 (β = 0.619, *p* = 0.002). Although VPA showed a significant effect at Wave 3 (β = 0.614, *p* = 0.002), its effect decreased at Wave 4 and was no longer significant (*p* = 0.444).

In the analysis of total cognitive ability (Figure 3c), MPA consistently demonstrated the most significant positive impact at each time point, especially at Wave 3 (β = 0.568, *p* = 0.004). This suggests that the protective effect of MPA on total cognition gradually increases over time. In contrast, the impact of LPA on total cognition remained relatively stable across waves, while the effect of VPA was relatively weaker, not reaching significance at Wave 4 (*p* = 0.501).

Overall, MPA showed the most significant and sustained protective effect on cognitive ability, particularly in episodic memory and mental intactness. VPA had some positive effect in the short term, but its long-term effect was less stable than that of MPA. The impact of LPA was smaller but stable, with its protective effect on cognitive ability improving over time.

Interestingly, cognitive ability naturally declined over time, especially in later waves such as Wave 3 and Wave 4, where the overall decline was more pronounced. This trend was evident in total cognition, episodic memory, and mental intactness. However, the introduction of PA seemed to delay this decline to some extent, particularly MPA, which exhibited a sustained significant protective effect on cognition.

### 3.4. Random Forest Model Prediction Results

In the prediction model for total cognitive ability, the mean squared error (MSE) was 9.08, indicating a relatively large model error. Variable importance analysis revealed that age had the most significant impact on total cognitive ability, far surpassing other factors, suggesting that age is the primary predictor of total cognition. Although PA intensity was included in the model, its influence was relatively small and did not rank highly in importance.

In the episodic memory model, the MSE was 2.70, indicating the best predictive performance among the models. Similarly to the total cognition model, age remained the most important predictor. Notably, the influence of PA intensity in this model became more prominent, rising in the variable importance ranking. This suggests that although PA intensity had a smaller impact in the overall cognition model, it may have a more significant protective effect on memory ability in the specific domain of episodic memory.

The mental intactness model had an MSE of 4.54, indicating moderate predictive performance. Consistently with other models, age had the most significant impact on mental intactness. While PA intensity was included, its influence was less than that of age, highlighting the dominant role of age in affecting mental intactness.

### 3.5. XGBoost Model Prediction Results

**Figure 4:**
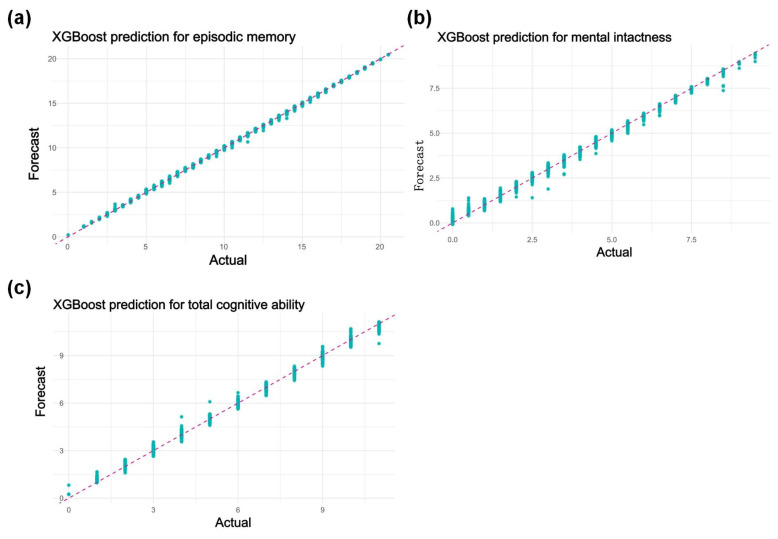
Fitting results of the XGBoost prediction model. (**a**) XGBoost prediction for episodic memory; (**b**) XGBoost prediction for mental intactness; (**c**) XGBoost prediction for total cognitive ability. In each figure, blue dots represent actual sample values, and the red dashed line represents the ideal where actual values equal predicted values. The closer the dots are to the line, the more accurate the model’s predictive ability.

As shown in Figure 4a, for episodic memory, the mean squared error (MSE) decreased steadily throughout the training process, with a final MSE of 0.0138. This indicates that the model performed well in predicting episodic memory, demonstrating a high level of accuracy. In Figure 4b, for mental intactness, the final MSE was 0.0157. The reduction in MSE reflects the model’s ability to effectively capture patterns in the data, leading to accurate predictions of mental intactness. In Figure 4c, for total cognitive ability, the MSE decreased consistently during training, reaching a final value of 0.0036. This significant reduction in error highlights the model’s robustness and predictive accuracy for total cognitive ability. To address overfitting concerns, we applied 5-fold cross-validation, L1 and L2 regularization, and hyperparameter tuning, resulting in a highly accurate model with low MSE values that effectively captured the relationship between physical activity intensity and cognitive function.

### 3.6. ARIMA Model Prediction of Future Trends in Cognitive Ability

**Figure 5:**
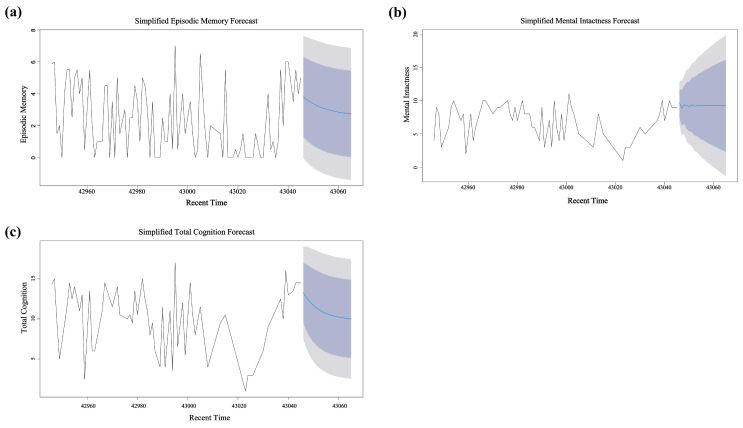
Prediction of future changes in cognitive ability using ARIMA models. (**a**) ARIMA prediction for episodic memory; (**b**) ARIMA prediction for mental intactness; (**c**) ARIMA prediction for total cognitive ability. In each figure, the blue line segment on the right represents the projected trend of the measured indicator over the foreseeable future.

As shown in Figure 5a, for episodic memory, the ARIMA(2,1,3) model had a Ljung–Box *p*-value of 0.097, indicating good model fit and high confidence in the prediction results. In the short term, episodic memory exhibited a stable downward trend.

In Figure 5b, for mental intactness, the ARIMA(1,1,2) model had a Ljung–Box *p*-value of 0.061, indicating a good fit. The prediction results suggest that mental intactness will remain relatively stable in the short term but may experience slight fluctuations and declines in the long term.

In Figure 5c, for total cognitive ability, the ARIMA(2,1,2) model had a Ljung–Box *p*-value of 0.102, suggesting good model fit. The prediction for total cognitive ability showed some volatility, with the model forecasting a continued slight downward trend in the future.

Overall, all models achieved statistical significance in terms of fit, indicating reliability in predicting changes in cognitive ability. In the short-term future, cognitive ability is expected to maintain the current trend and level; that is, the significant relationship between PA intensity and cognitive ability will persist. If individuals maintain or slightly increase their current level of PA intensity, cognitive ability may remain higher, offsetting the natural decline associated with aging.

## 4. Discussion

In conclusion, our study provides further evidence supporting the protective effects of PA on the cognitive function of middle-aged and elderly individuals. More importantly, we distinguished between different exercise intensities and identified varying outcomes associated with each. Both LPA and MPA demonstrated significant correlations with cognitive preservation and exhibited long-term effects, whereas VPA did not show such benefits. These findings were further validated through machine learning models. Although numerous studies have suggested an association between physical activity (PA) and the preservation of cognitive function, findings have been inconsistent. For instance, a meta-analysis of longitudinal studies indicated that higher levels of PA are linked to a reduced risk of cognitive decline (relative risk [RR] = 0.65; 95% confidence interval [CI]: 0.55–0.76). Similarly, another meta-analysis focusing on long-term follow-up studies found that PA is significantly associated with overall cognition (standardized regression coefficient = 0.03; 95% CI: 0.02–0.03) and changes in overall cognitive function (standardized regression coefficient = 0.01; 95% CI: 0.01–0.02). However, some studies report conflicting results. For example, the Whitehall II prospective cohort study found no significant association between PA and cognitive decline [9].

Clarifying the roles of different PA intensities remains controversial. Some research suggests that LPA offers more stable cognitive protective effects over long-term follow-up, especially among middle-aged and elderly individuals. However, these studies often rely on self-reported PA intensity, involve small sample sizes, and have relatively short follow-up periods [25,26]. Conversely, intervention studies on high-intensity interval training (HIIT) have focused on the short-term effects of vigorous-intensity PA on executive function and working memory, primarily in younger populations or elderly individuals with good cardiovascular health [27]. The relationship between PA intensity and cognitive improvement may be influenced by factors such as age, health status, and the type of PA. These findings highlight the complexity of PA intensity’s impact on cognitive function, complicating the establishment of standardized guidelines. The 2018 Physical Activity Guidelines noted the difficulty in ranking the effects of different PA intensities on cognitive enhancement, emphasizing the need for more scientific methods to confirm PA intensity’s influence on cognitive function [28]. Based on our findings, MPA demonstrated significant advantages in cognitive protection among middle-aged and elderly individuals. While LPA also showed some protective effects, MPA is clearly more advisable.

To objectively and comprehensively evaluate the long-term effects of PA and its intensity, we introduced machine learning models to verify MPA’s advantages in sustained cognitive protection and to analyze its robustness. Both machine learning methods validated the effectiveness of MPA in safeguarding cognitive function, especially in the XGBoost model, which achieved a highly precise fit.

To further elucidate PA’s long-term protective effects on cognitive function, we innovatively employed the ARIMA model to predict future trends in cognitive changes. The ARIMA model not only captured the immediate impact of different PA intensities on cognitive function but also revealed potential future trajectories. The predictions indicated that MPA has significant advantages in the long-term maintenance of cognitive ability, confirming that PA can continue to provide protective effects over time. This finding offers a basis for developing long-term health intervention strategies, suggesting that future efforts to delay cognitive decline in middle-aged and elderly populations should focus more on appropriate PA programs. Overall, the application of the ARIMA model enhances the prospective value of our study.

In terms of cognitive protection, PA may exert its effects through multiple physiological mechanisms. Firstly, PA can significantly elevate levels of brain-derived neurotrophic factor (BDNF), particularly in the hippocampus and prefrontal cortex—brain regions linked to memory and executive function [29]. Elevated BDNF levels promote neuronal survival and synaptic plasticity [30]. Additionally, PA can enhance cerebral blood flow, improving oxygen and glucose supply—particularly beneficial for elderly individuals with impaired executive function [31,32]. This process is accompanied by the production of endothelial nitric oxide (NO) [33], which promotes vasodilation and the clearance of metabolic waste, ultimately benefiting neural health [34]. From an inflammation regulation perspective, PA can reduce chronic systemic inflammation by inhibiting pro-inflammatory cytokines (e.g., IL-6, TNF-α [35]) and increasing anti-inflammatory cytokines (e.g., IL-10) [36], thereby protecting the nervous system from inflammation-induced damage [37]. Reducing inflammation helps slow neurodegenerative changes, such as early pathological alterations in Alzheimer’s disease [38].

The long-term efficacy of MPA in cognitive protection may be explained by its sustainability [39]. Compared to VPA, MPA is more manageable, avoiding excessive fatigue and post-exercise discomfort, which makes it easier to maintain over the long term [40]. Studies have shown that regular physical activity is crucial for cognitive protection. Although VPA can significantly improve cardiorespiratory function in the short term, it is challenging to sustain and may impose a greater physical burden on the elderly, reducing adherence [41,42]. Furthermore, MPA provides continuous cerebral blood flow and nutritional support; long-term stable oxygen supply and metabolic waste clearance are vital for maintaining cognitive function [43]. Combined with sustained elevation of BDNF and promotion of neural plasticity, MPA can protect against and delay cognitive decline through long-term regulatory effects. Importantly, in middle-aged and elderly individuals, the intense stress response associated with VPA may lead to higher levels of cortisol and oxidative stress [44], potentially inhibiting long-term neural plasticity.

While our study has made strides in exploring the protective effects of different PA intensities on cognitive function in middle-aged and elderly individuals—particularly through the innovative use of longitudinal data and the ARIMA model for future trend prediction—it has limitations. First, the sample was drawn from a longitudinal survey of Chinese elderly individuals, which may limit the generalizability of the findings to other regions. Secondly, PA intensity was based on self-reported data rather than objective measurements. Lastly, as a preliminary exploration, we only utilized the ARIMA time-series prediction model. It is worth noting that ARIMA has wide confidence intervals for its predictions, but this reflects the high volatility of the data and the uncertainty in the model’s predictions. Nevertheless, the predictions are still significant as they reveal a trend towards protection of long-term cognitive function by physical activity. This trend, despite the uncertainty, provides valuable evidence that moderate-intensity physical activity may have a sustained protective effect in delaying cognitive decline. More importantly, while our findings highlight the significance of physical activity for cognitive function, it is equally essential not to overlook the need to reduce sedentary behavior. Our previous research demonstrated that decreasing sedentary time contributes to the well-being of middle-aged and elderly, benefiting not only bone health but also psychological, cognitive, and cardiovascular health [45,46]. Similarly, other studies have emphasized that balancing physical activity and sedentary behavior throughout the day plays a crucial role in promoting overall health in elderly [47], extending beyond cognitive function alone. Therefore, focusing solely on physical activity without considering the reduction in sedentary behavior provides an incomplete perspective.

Future research should focus on larger samples and longer time spans, possibly incorporating multi-regional datasets to enhance the generalizability of results. Objective measurement tools like accelerometers [48] could improve the accuracy of PA intensity assessments. Additionally, employing various time-series models, such as Latent Class Trajectory Model (LCTM) networks [49], could further validate our findings. It is also recommended that future experimental studies control for activity types to validate the longitudinal trends and effectiveness of LPA, MPA, and VPA observed during controlled experiments.

## 5. Conclusions

This study provides preliminary evidence that both LPA and MPA are associated with the preservation of cognitive function in middle-aged and elderly, with MPA showing more substantial protective effects. In contrast, VPA did not exhibit significant benefits. These findings were validated using the random forest and XGBoost algorithms. Furthermore, according to ARIMA forecasts, the cognitive protective effects of MPA and LPA, rather than VPA, are likely to persist over the long term.

## Figures and Tables

**Table 1:** Baseline characteristics of participants by physical activity level (*n* = 5206).

Characteristic	HPA (*n* = 1777)	MPA (*n* = 1621)	LPA (*n* = 1300)	NE (*n* = 508)	*p*-Value
Age, (SD)	56.22 (8.37)	57.56 (9.16)	60.59 (9.90)	59.53 (10.21)	<0.001
Episodic memory (SD)	3.68 (1.59)	3.91 (1.67)	3.75 (1.67)	3.50 (1.70)	<0.001
Executive function (SD)	8.11 (2.51)	8.60 (2.44)	8.44 (2.52)	7.96 (2.78)	<0.001
Total cognition (SD)	11.79 (3.39)	12.52 (3.42)	12.19 (3.56)	11.45 (3.80)	<0.001
Female, *n* (%)	752 (42.3)	932 (57.5)	711 (54.7)	285 (56.1)	<0.001
Male, *n* (%)	1025 (57.7)	689 (42.5)	589 (45.3)	223 (43.9)	
Married, *n* (%)	1637 (92.1)	1455 (89.8)	1101 (84.7)	451 (88.8)	<0.001
Rural residence, *n* (%)	452 (25.4)	766 (47.3)	768 (59.1)	227 (44.7)	<0.001
Hypertension, *n* (%)	338 (19.0)	431 (26.6)	411 (31.6)	152 (29.9)	<0.001
Diabetes, *n* (%)	60 (3.4)	106 (6.5)	122 (9.4)	45 (8.9)	<0.001
Cancer, *n* (%)	15 (0.8)	15 (0.9)	16 (1.2)	6 (1.2)	0.706
Lung disease, *n* (%)	162 (9.1)	160 (9.9)	131 (10.1)	56 (11.0)	0.589
Heart disease, *n* (%)	125 (7.0)	233 (14.4)	225 (17.3)	76 (15.0)	<0.001
Arthritis, *n* (%)	585 (32.9)	542 (33.4)	397 (30.5)	156 (30.7)	0.295
Dyslipidemia, *n* (%)	122 (6.9)	194 (12.0)	199 (15.3)	51 (10.0)	<0.001
Liver disease, *n* (%)	69 (3.9)	46 (2.8)	38 (2.9)	18 (3.5)	0.3
Kidney disease, *n* (%)	98 (5.5)	88 (5.4)	74 (5.7)	29 (5.7)	0.989
Digestive disease, *n* (%)	430 (24.2)	357 (22.0)	274 (21.1)	83 (16.3)	0.002
Asthma, *n* (%)	62 (3.5)	70 (4.3)	65 (5.0)	27 (5.3)	0.13
Current drinker, *n* (%)	828 (46.6)	622 (38.4)	452 (34.8)	181 (35.6)	<0.001
Current smoker, *n* (%)	818 (46.0)	557 (34.4)	473 (36.4)	186 (36.6)	<0.001
Education, *n* (%)					<0.001
- Below primary school	752 (42.3)	591 (36.5)	481 (37.0)	218 (42.9)	
- Primary school	445 (25.0)	356 (22.0)	291 (22.4)	101 (19.9)	
- Middle school	424 (23.9)	379 (23.4)	294 (22.6)	126 (24.8)	
- High school and above	156 (8.8)	295 (18.2)	234 (18.0)	63 (12.4)	

Note: HPA: high-intensity physical activity; MPA: moderate-intensity physical activity; LPA: low-intensity physical activity; NE: no physical activity.

## Data Availability

Data can be found online at: http://charls.pku.edu.cn/.

## Supplementary Materials

The following supporting information can be downloaded at: https://www.mdpi.com/article/10.3390/life14101343/s1. Table S1. Stepwise regression results. Table S2. Temporal interaction term analysis. Table S3. Subgroup analysis. Table S4. ARIMA. Figure S1. Sensitivity analysis. Figure S2. Randomized forest prediction maps for Episodic memory. Figure S3. Randomized forest prediction maps for mental intactness. Figure S4. Randomized forest prediction maps for total cognition. Figure S5. Randomized forest for episodic memory. Figure S6. Randomized forest for mental intactness. Figure S7. Randomized forest for total cognition.
