# Supplementary material for: Evidence of the Long-Term Protective Effect of Moderate-Intensity Physical Activity on Cognitive Function in Middle-Aged and Elderly Individuals: A Predictive Analysis of Longitudinal Studies
Source: Life (Basel). 2024 Oct 21;14(10):1343. doi: 10.3390/life14101343 (PMC11509916; doi:10.3390/life14101343)
Supplement: Supplementary file 1 [file life-14-01343-s001.zip › life-3263984-supplementary.pdf]

Table S1 Stepwise regression results
Table S2 Temporal interaction term analysis
Table S3 Subgroup analysis
Table S4 ARIMA
Figure S1 sensitive analysis
Figure S2 Randomized forest prediction maps for Episodic memory
Figure S3 Randomized forest prediction maps for mental intactness
Figure S4 Randomized forest prediction maps for total cognition
Figure S5 Randomized forest for Episodic memory
Figure S6 Randomized forest for mental intactness
Figure S7 Randomized forest for total cognition

Table S1. Stepwise regression results

**Physical activity**	**Cognition Domain**	**Model 1 β**	**Model 1 95% CI**	**Model 1 P value**	**Model 2 β**	**Model 2 95% CI**	**Model 2 P value**	**Model 3 β**	**Model 3 95% CI**	**Model 3 P value**
Low	Global cognition	0.271	0.178–0.912	0.004	0.21	0.119–0.993	0.013	0.207	0.176–0.987	0.012
Moderate	Global cognition	0.486	0.514–1.224	< 0.001	0.348	0.317–1.171	0.001	0.345	0.314–1.164	0.001
Vigorous	Global cognition	0.317	–0.153–0.553	0.267	0.247	–0.187–0.667	0.272	0.249	–0.189–0.671	0.271
Low	Episodic memory	0.314	0.065–0.441	0.008	0.216	0.005–0.457	0.048	0.218	0.004–0.454	0.046
Moderate	Episodic memory	0.534	0.194–0.558	< 0.001	0.401	0.093–0.539	0.005	0.404	0.092–0.538	0.005
Vigorous	Episodic memory	0.345	–0.039–0.321	0.128	0.222	–0.113–0.333	0.336	0.229	–0.114–0.335	0.335
Low	Mental intactness	0.499	0.029–0.555	0.029	0.394	0.007–0.643	0.045	0.394	0.006–0.642	0.044
Moderate	Mental intactness	0.898	0.238–0.748	< 0.001	0.707	0.117–0.741	0.007	0.708	0.116–0.740	0.007
Vigorous	Mental intactness	0.611	–0.194–0.312	0.646	0.455	–0.182–0.442	0.413	0.463	–0.183–0.443	0.412

Table S2. Temporal interaction term analysis

Variable	Model 1 Estimate	Model 1 Std.Error	Model 1 P value	Model 2 Estimate	Model 2 Std.Error	Model 2 P value	Model 3 Estimate	Model 3 Std.Error	Model 3 P value
(Intercept)	11.8595	0.1258	<0.0001	11.35	0.2649	<0.0001	11.44	0.2674	<0.0001
wave2	-0.2777	0.1861	0.1356	-0.083	0.1807	0.6458	-0.067	0.1815	0.7115
wave3	-0.9272	0.1812	3.16E-07	-0.648	0.1726	0.0002	-0.667	0.1762	0.0002
wave4	-0.6337	0.1788	0.0004	-0.239	0.1706	0.1617	-0.263	0.1714	0.1254
Activity_Level Low	0.334	0.1455	0.0218	0.351	0.1383	0.0112	0.357	0.1384	0.0099
Activity_Level Moderate	0.5489	0.141	9.92E-05	0.45	0.1341	0.0008	0.45	0.1342	0.0008
Activity_Level Vigorous	0.2719	0.1398	0.0517	0.19	0.1331	0.1539	0.186	0.1333	0.1623
wave2 Low	0.2418	0.2213	0.2748	0.132	0.2144	0.5384	0.104	0.2154	0.6306
wave3 Low	0.3533	0.2152	0.1007	0.271	0.2045	0.1859	0.293	0.2086	0.1595
wave4 Low	0.1685	0.2062	0.4138	-0.008	0.1951	0.967	-0.024	0.1953	0.9001
wave2 Moderate	0.3229	0.2136	0.1306	0.225	0.2071	0.2774	0.213	0.2081	0.3063
wave3 Moderate	0.6822	0.2076	0.001	0.615	0.1975	0.0019	0.619	0.201	0.0021
wave4 Moderate	0.4195	0.2016	0.0375	0.326	0.1909	0.0874	0.312	0.1911	0.1027
wave2 Vigorous	0.4368	0.2091	0.0368	0.309	0.2032	0.1284	0.301	0.2043	0.1403
wave3 Vigorous	0.6864	0.2041	0.0008	0.568	0.1944	0.0035	0.614	0.198	0.0019
wave4 Vigorous	0.2772	0.2001	0.1659	0.128	0.1898	0.5011	0.145	0.1901	0.4442

Table S3. Subgroup analysis

subgroups	Activity Level	Cognition Domain	Estimate	Std. Error	95% CI	P value
Female	Low	Memory	0.305	0.0667	0.174–0.436	4.68E-06
Female	Moderate	Memory	0.565	0.0637	0.444–0.686	4.38E-14
Female	High	Memory	0.308	0.0679	0.175–0.441	5.61E-06
Female	Low	Executive Function	0.345	0.083	0.182–0.508	3.39E-05
Female	Moderate	Executive Function	0.597	0.0794	0.442–0.752	6.13E-14
Female	High	Executive Function	0.228	0.0853	0.061–0.395	0.00743
Female	Low	Total Cognition	0.525	0.1157	0.298–0.752	5.81E-06
Female	Moderate	Total Cognition	1.008	0.1106	0.791–1.225	3.27E-12
Female	High	Total Cognition	0.486	0.1192	0.251–0.721	4.56E-05
Male	Low	Memory	0.232	0.0653	0.104–0.360	0.00038
Male	Moderate	Memory	0.377	0.0658	0.248–0.506	9.89E-09
Male	High	Memory	0.315	0.0642	0.189–0.441	9.54E-07
Male	Low	Executive Function	0.258	0.0777	0.106–0.410	0.0009
Male	Moderate	Executive Function	0.505	0.0783	0.352–0.658	1.25E-10
Male	High	Executive Function	0.329	0.0767	0.179–0.479	1.84E-05
Male	Low	Total Cognition	0.434	0.1115	0.215–0.653	0.000102
Male	Moderate	Total Cognition	0.785	0.1126	0.564–1.006	3.27E-12
Male	High	Total Cognition	0.609	0.1104	0.391–0.827	3.54E-08
Under 60	Low	Memory	0.256	0.0714	0.118–0.394	0.000336
Under 60	Moderate	Memory	0.329	0.0681	0.195–0.463	1.31E-06

Under 60	High	Memory	0.119	0.0682	-0.014–0.252	0.08011
Under 60	Low	Executive Function	0.109	0.0861	-0.059–0.277	0.2065
Under 60	Moderate	Executive Function	0.212	0.0821	0.051–0.374	0.00965
Under 60	High	Executive Function	-0.042	0.0827	-0.204–0.120	0.6088
Under 60	Low	Total Cognition	0.289	0.121	0.052–0.526	0.0168
Under 60	Moderate	Total Cognition	0.455	0.115	0.227–0.683	8.17E-05
Under 60	High	Total Cognition	0.063	0.116	-0.165–0.291	0.5895
60 and Over	Low	Memory	0.348	0.0615	0.229–0.467	1.57E-08
60 and Over	Moderate	Memory	0.596	0.0614	0.474–0.718	1.56E-15
60 and Over	High	Memory	0.327	0.0633	0.204–0.450	2.51E-07
60 and Over	Low	Executive Function	0.527	0.0785	0.373–0.681	2.02E-11
60 and Over	Moderate	Executive Function	0.798	0.0785	0.644–0.952	2.00E-16
60 and Over	High	Executive Function	0.563	0.0816	0.403–0.723	5.61E-12
60 and Over	Low	Total Cognition	0.781	0.111	0.563–0.999	1.79E-12
60 and Over	Moderate	Total Cognition	1.277	0.111	1.059–1.495	2.00E-16
60 and Over	High	Total Cognition	0.865	0.115	0.637–1.093	6.43E-14
Healthy	Low	Memory	0.27	0.0976	0.078–0.462	0.00568
Healthy	Moderate	Memory	0.501	0.0945	0.316–0.686	1.18E-07
Healthy	High	Memory	0.235	0.0943	0.050–0.420	0.01264
Healthy	Low	Executive Function	0.438	0.1206	0.202–0.674	0.000289

Healthy	Moderate	Executive Function	0.615	0.1167	0.388–0.842	1.42E-07
Healthy	High	Executive Function	0.299	0.1172	0.069–0.529	0.010676
Healthy	Low	Total Cognition	0.601	0.1712	0.265–0.937	0.000452
Healthy	Moderate	Total Cognition	0.981	0.1655	0.657–1.305	3.35E-09
Healthy	High	Total Cognition	0.478	0.1667	0.152–0.804	0.004159
Unhealthy	Low	Memory	0.28	0.0537	0.174–0.386	1.92E-07
Unhealthy	Moderate	Memory	0.5	0.0528	0.396–0.604	< 2e-16
Unhealthy	High	Memory	0.338	0.054	0.232–0.444	3.92E-10
Unhealthy	Low	Executive Function	0.32	0.0664	0.189–0.451	1.45E-06
Unhealthy	Moderate	Executive Function	0.561	0.0653	0.433–0.689	< 2e-16
Unhealthy	High	Executive Function	0.368	0.0673	0.236–0.500	4.47E-08
Unhealthy	Low	Total Cognition	0.518	0.0936	0.335–0.701	3.23E-08
Unhealthy	Moderate	Total Cognition	0.943	0.0921	0.763–1.123	< 2e-16
Unhealthy	High	Total Cognition	0.658	0.0951	0.471–0.845	4.97E-12

Table S4. ARIMA

Cognition Domain	Model	Parameter	Estimate	Std. Error	AIC	BIC
Total Cognition	ARIMA(2,1,2)	ar1	0.3545	0.3327	171002	171080
		...	...	...		
Episodic Memory	ARIMA(2,1,3)	ar1	0.592	0.1199	168258	168318.7
		...	...	...		
Mental Intactness	ARIMA(1,1,2)	ar1	0.3647	0.0285	191678.5	191713.1
		...	...	...		

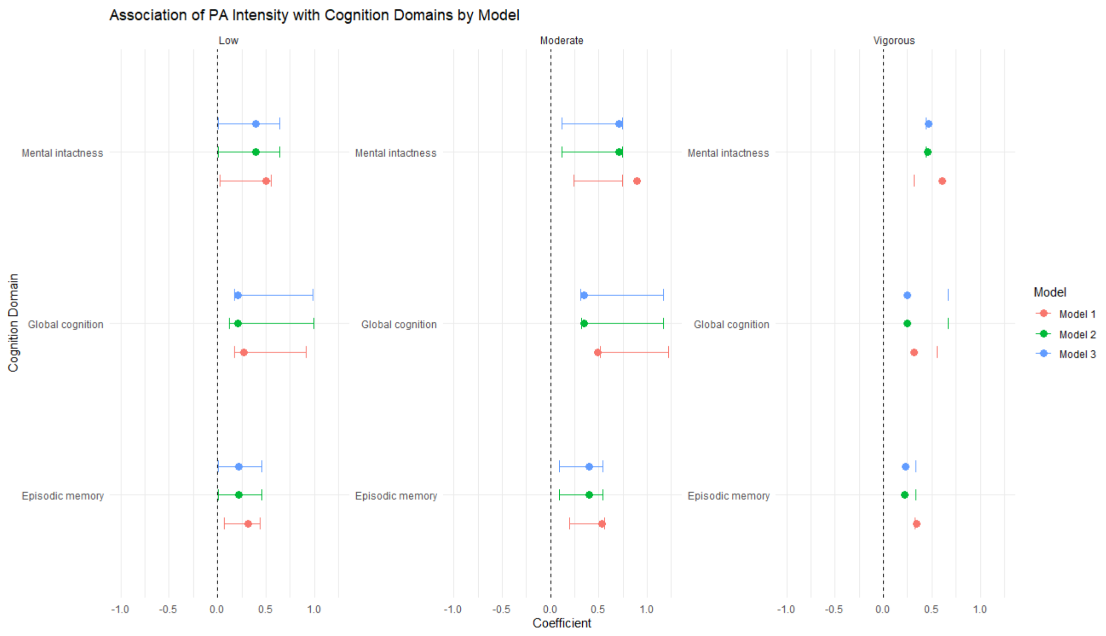

Figure S1. sensitive analysis

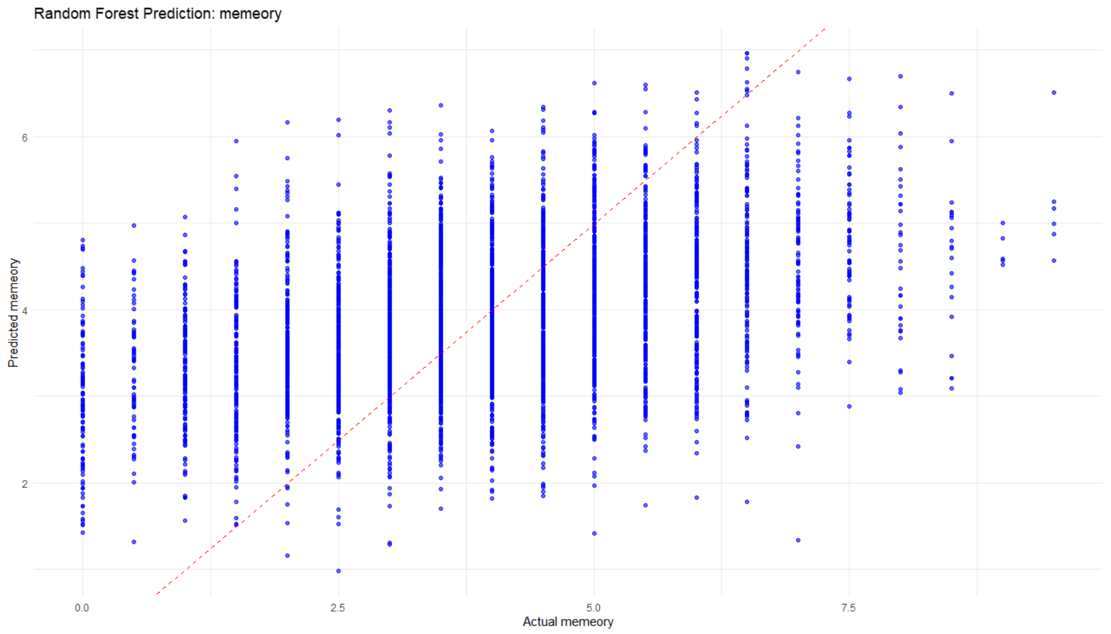

Figure S2. Randomized forest prediction maps for situational memory

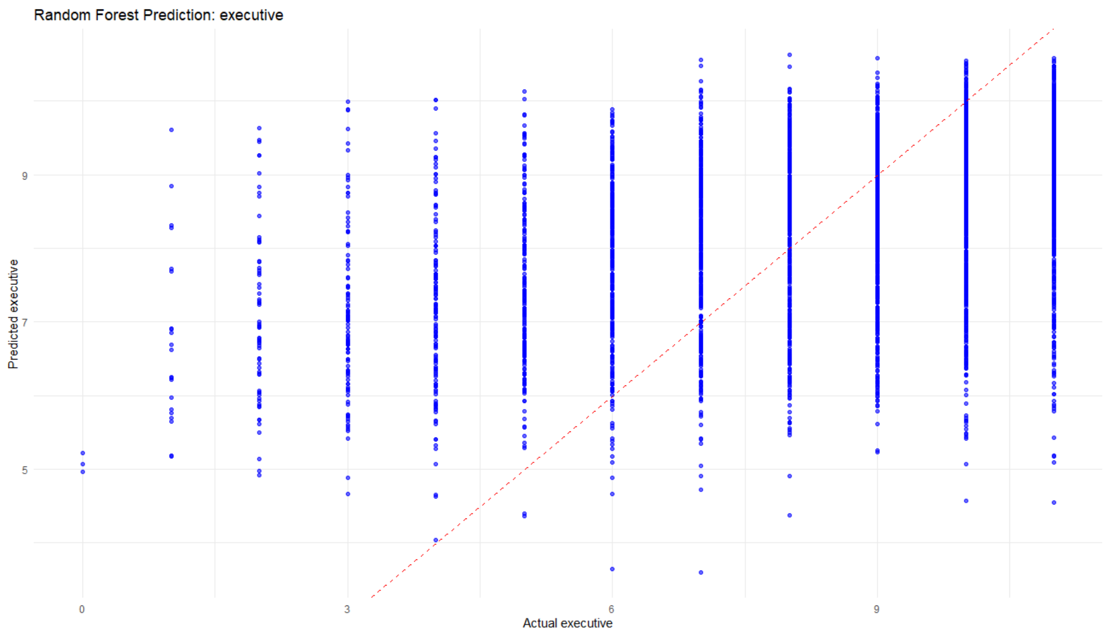

Figure S3. Randomized forest prediction maps for excutive

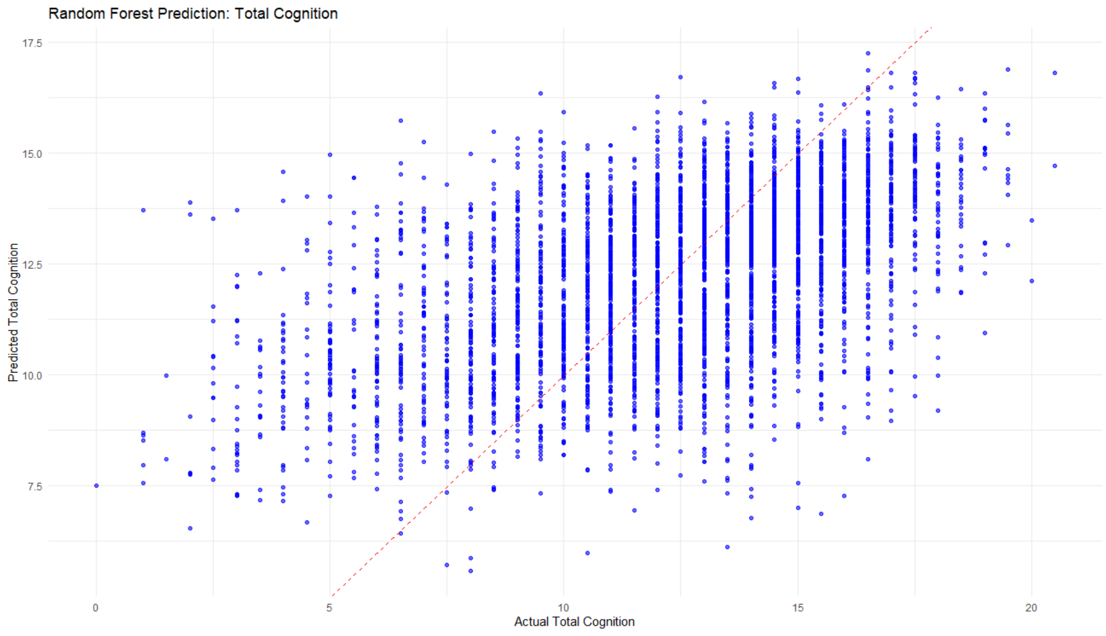

Figure S4. Randomized forest prediction maps for total cognition

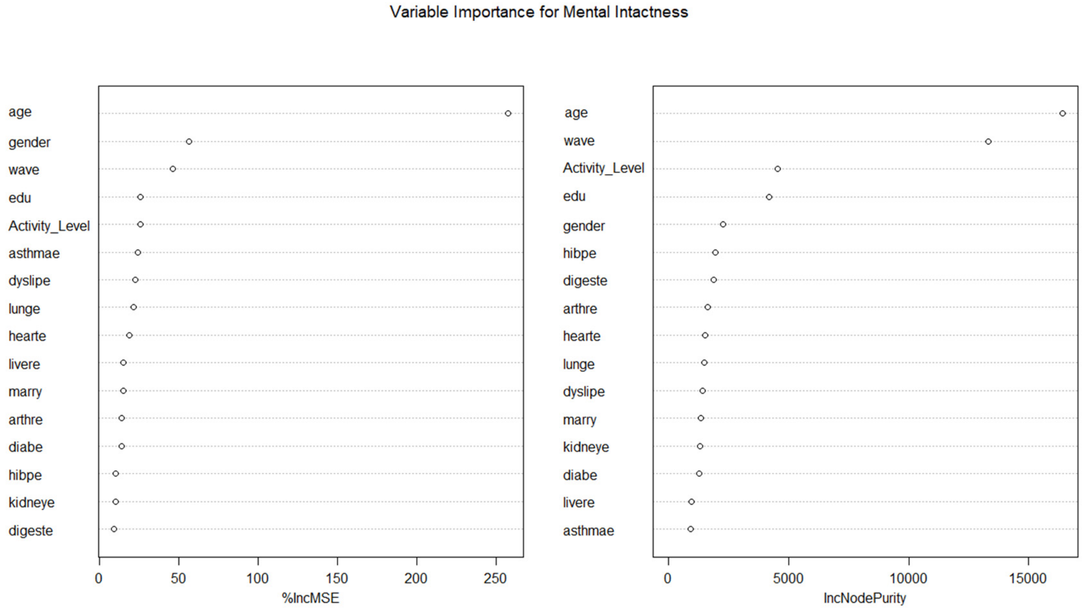

Figure S5. Randomized forest for mental intactness

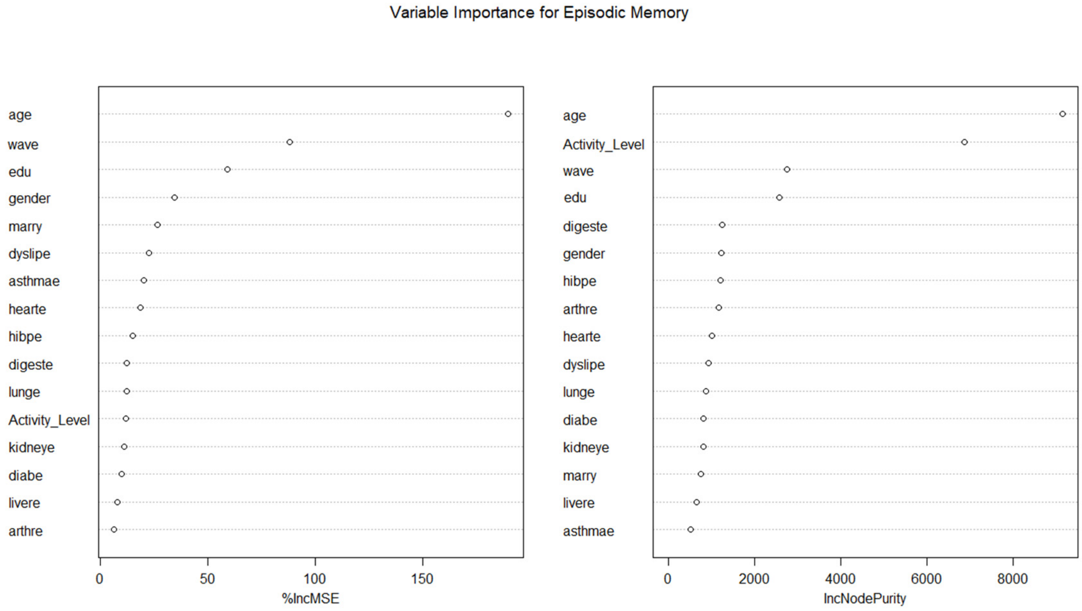

Figure S6. Randomized forest for episodic memory

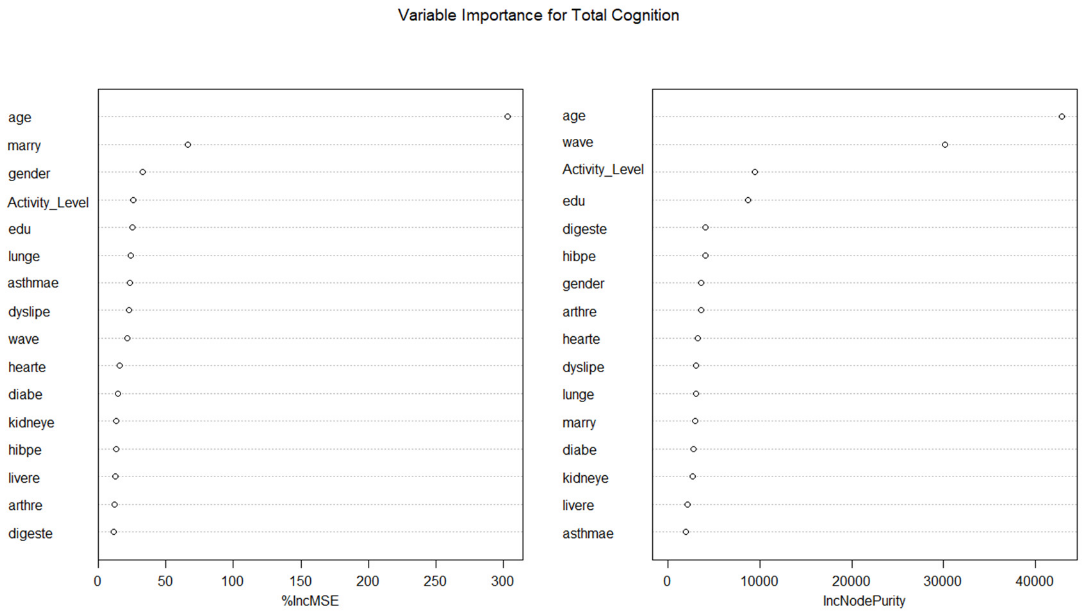

Figure S7. Randomized forest for total cognition
